# Laryngeal Diphtheria, Treated by Antitoxin and Calomel Fumigation*Case reported before the Richmond Academy of Medicine and Surgery, June 8, 1897.

**Published:** 1897-07

**Authors:** E. C. Levy

**Affiliations:** Professor of Histology, Pathology, and Bacteriology at the Medical College of Virginia; 813 Floyd Avenue


### LARYNGEAL DIPHTHERIA, TREATED BY ANTITOXIN AND CALOMEL FUMIGATION.*

BY E. C. LEVY, M. D.

Professor of Histology, Pathology, and Bacteriology at the Medical College of Virginia.

I wish to report this case, not because it presents any remarkable phases to those of you who have availed yourselves of the remedies which were used in its treatment, but merely to illustrate how the advent of these additions to our means of combating this dread disease has revolutionized its treatment. So strong a statement as this would not be justifiable were it the outcome of the observation of a single case, but I make it confidently because the result in the case I wish to report in conformity with my previous experience in a large number of

* Case reported before the Richmond Academy of Medicine and Surgery, June 8, 1897.

cases at the Willard Parker Hospital, New York, and with the reports of thousands of cases treated by others during the past few years.

Gladys E., age 4 years 6 months, was seen by Dr. Upshur on the morning of Friday, May 28th. The following history was obtained from the parents. Previous history, negative. Had been suffering with a slight "cold" for three days. On night of 26th had a mild attack of croup. Was much better the next day, but complained of throat hurting her. Had another attack of croup on night of Thursday, 27th, which did not improve on the following day. From time of first attack of croup child was drowsy and languid. On morning of Friday, the 28th, the parents took her to a neighboring drugstore for advice, but to the credit of this member of a much-abused fraternity, the druggist's only advice was to send for a doctor.

When Dr. Upshur saw the case there was marked obstructive dyspnœa, and a small patch of pseudo-membrane on the left tonsil. These symptoms led the doctor to diagnose the case as one of diphtheria, and he prescribed a mixture of Tr. Ferri. Chlor. Hydrarg. Chlor. Cor., and Glycerine. In the afternoon he sent for me to take a culture. By this time the croup symptoms were much more pronounced, and the pseudo-membrane above had spread rapidly, both tonsils and portions of the soft palate being covered with a thick yellow deposit. At 9 P. M. Dr. Upshur telephoned for me to see the case in consultation. I advised the immediate use of antitoxin. As Dr. Upshur was to leave town on the following day he requested me to take charge of the case. I at once ordered the neck poulticed, and injected 1800 units of antitoxin as soon as it could be procured, continuing the mixture which Dr. Upshur had prescribed. (I injected at the same time 200 units into the two-year old sister of the patient, who had been into the same room, and whom it was impossible to isolate.) During the early part of the night there was considerable dyspnœa, with retraction of the supra-sternal notch and supra-clavicular regions and I was prepared to intubate should it become necessary. Later on the dyspnœa became less, and I left Dr. Dorsey Morris in charge for the night. On seeing the child the following morning (Saturday) Dr. Morris reported that she had passed a comparatively comfortable night, and had coughed up a good size piece of membrane. During the day she continued to do well. The culture taken on the preceding day showed an abundant growth of Klebs-Loeffler bacilli. At 4:30 P. M. I injected 1000 units more of antitoxin. At midnight I again saw the child and found her suffering with urgent dyspnœa, which, the nurse reported, had begun to appear about 9 o'clock. I discontinued the iron and bichloride mixture, and ordered a mixture of syrup of ipecac and compound syrup of squills to be given every hour to the point of nausea.

I was sent for hurriedly four hours later and found the child much worse. Retraction was now marked, the entire sternum sinking in at each inspiration, and there was beginning cyanosis. The necessity for giving relief was imperative. I had the instruments all prepared for intubation, but decided, before resorting to operative measures, which always increase the gravity of a case, especially in private practice, to try calomel fumigation, believing as I did that there was present a considerable spasmodic element. An improvised tent was accordingly rigged up over the child and twenty grains of calomel sublimated under it. In five minutes improvement could be noticed; in fifteen minutes all urgent symptoms had subsided, and in half an hour the child was asleep and breathing so quietly that her respirations could not be heard across the room. On paying my afternoon call I found the child sitting up in bed, playing with her doll and begging, with quite a loud voice, to be allowed to sit at the table. On the following day I began the administration of Strych. Sulp. gr. 1-128 four times a day. The child has made an uninterrupted recovery. The membrane disappeared from the throat on last Wednesday, the 2d, but the bacilli are still persistent. The little sister of the patient, to whom I gave 200 units of antitoxin, has been in the sick-room the entire time but has developed no symptoms of the disease.

This is the third case of laryngeal diphtheria I have seen in consultation during the past six months. The other two I had the honor of reporting to the Academy on a previous occasion. The first of these cases was not a very severe one, although the dyspnœa was very urgent at times, and the child made a prompt recovery under antitoxin, general sustaining treatment, and steam inhalations. The second was a case seen in Manchester in consultation with Dr. Mathews. This case required intubation at once, and died sixteen hours after I first saw it, from extension of membrane far down into the trachea.

The case I have reported here to-night is a most instructive one, and illustrates forcibly the advantages of antitoxin treatment. Those who oppose this remedy must show us equal results obtained by other methods before any theoretical objections to its use will have weight. It also illustrates the point to which I have directed attention before on the floor of the Academy, viz.: that the use of antitoxin does not justify us in neglecting a single one of the other measures which time and experience have shown to be of benefit. In this case, had the other remedies, especially the calomel fumigation, not been resorted to, I believe the child would not have recovered. On the other hand, had antitoxin injections not been employed I am confident that all the other measures would have been unavailing. Any plan of treatment which, within forty hours, may convert a serious case of a most serious disease, laryngeal diphtheria, into one in which a favorable prognosis can be given must commend itself to the consideration of every thoughtful physician.

*813 Floyd Avenue.*

### A DECISION OF IMPORTANCE TO PHYSICIANS.

A case of interest to physicians generally was decided by Judge Dunbar, at Boston, last week. The circumstances of the case, as reported, were that previous to May 1, 1896, Dr. Oscar F. George had a lucrative practice in Lynn. On that date he sold it to Dr. Edward B. Herrick, who came from Amherst, Mass., signing an agreement not to practice in the city as long as Dr. Herrick remained there. He then went to Newburyport and later to Vermont, and about March 1, 1897, came to Swampscott, where he located and resumed practice, and, as he admitted upon the witness-stand, again began practice among his old patients in Lynn. Dr. Herrick brought a bill in equity in the Superior Court against Dr. George, to have him restrained from practicing in Lynn. As a result Judge Dunbar enjoined the defendant from practicing in Lynn in violation of his promise. The decision is important from the fact that, while the defendant admitted that morally he was bound to keep his agreement, legally he was not so bound. The judge, however, decided that he was both legally and morally bound to keep his agreement, and enjoined the defendant from further tresspassing upon the ground to which he had signed away all claim.—*Boston Med. and Surg. Journal.*

